# Proteomics-Based Identification of Dysregulated Proteins in Breast Cancer

**DOI:** 10.3390/proteomes10040035

**Published:** 2022-10-21

**Authors:** Anca-Narcisa Neagu, Madhuri Jayathirtha, Danielle Whitham, Panashe Mutsengi, Isabelle Sullivan, Brindusa Alina Petre, Costel C. Darie

**Affiliations:** 1 Laboratory of Animal Histology, Faculty of Biology, “Alexandru Ioan Cuza” University of Iasi, Carol I bvd, No. 20A, 700505 Iasi, Romania; 2 Biochemistry & Proteomics Laboratories, Department of Chemistry and Biomolecular Science, Clarkson University, Potsdam, NY 13699-5810, USA; 3 Laboratory of Biochemistry, Faculty of Chemistry, “Alexandru Ioan Cuza” University of Iasi, Carol I bvd, No. 11, 700506 Iasi, Romania

**Keywords:** breast cancer, proteomics, dysregulated proteins, proteoforms

## Abstract

Immunohistochemistry (IHC) is still widely used as a morphology-based assay for in situ analysis of target proteins as specific tumor antigens. However, as a very heterogeneous collection of neoplastic diseases, breast cancer (BC) requires an accurate identification and characterization of larger panels of candidate biomarkers, beyond ER, PR, and HER2 proteins, for diagnosis and personalized treatment, without the limited availability of antibodies that are required to identify specific proteins. Top-down, middle-down, and bottom-up mass spectrometry (MS)-based proteomics approaches complement traditional histopathological tissue analysis to examine expression, modification, and interaction of hundreds to thousands of proteins simultaneously. In this review, we discuss the proteomics-based identification of dysregulated proteins in BC that are essential for the following issues: discovery and validation of new biomarkers by analysis of solid and liquid/non-invasive biopsies, cell lines, organoids and xenograft models; identification of panels of biomarkers for early detection and accurate discrimination between cancer, benign and normal tissues; identification of subtype-specific and stage-specific protein expression profiles in BC grading and measurement of disease progression; characterization of new subtypes of BC; characterization and quantitation of post-translational modifications (PTMs) and aberrant protein–protein interactions (PPI) involved in tumor development; characterization of the global remodeling of BC tissue homeostasis, diagnosis and prognostic information; and deciphering of molecular functions, biological processes and mechanisms through which the dysregulated proteins cause tumor initiation, invasion, and treatment resistance.

## 1. Introduction

Breast cancer (BC) represents a group of neoplastic diseases that emphasize a high intratumoral and intertumoral heterogeneity [1]. In the actual era of predictive, preventive, personalized, precision, and participatory medicine (“P5”) [2,3,4,5], the holistic investigation in oncobreastomics research converges towards the discovery and validation of specific panels of multi-“omics” tumor biomarkers for diagnosis, prognosis, staging/grading, treatment assessment, or measurement of disease progression and finding new targets for cancer treatment strategies. Consequently, the panels of biomarkers offer a better clinical information compared with any single marker from the panel [6]. Proteomics characterization of breast tumors is essential for understanding of molecular aberrations, especially based on signatures of cancer-associated proteins (CAPs), which are known as a distinct group of potential biomarkers linked to cancer [7], that by their loss, downregulated or overexpressed level or by their PTMs and aberrant PPIs may contribute to the dysregulated cellular functions, tumor development, and patient survival [8]. 

**Figure 1:**
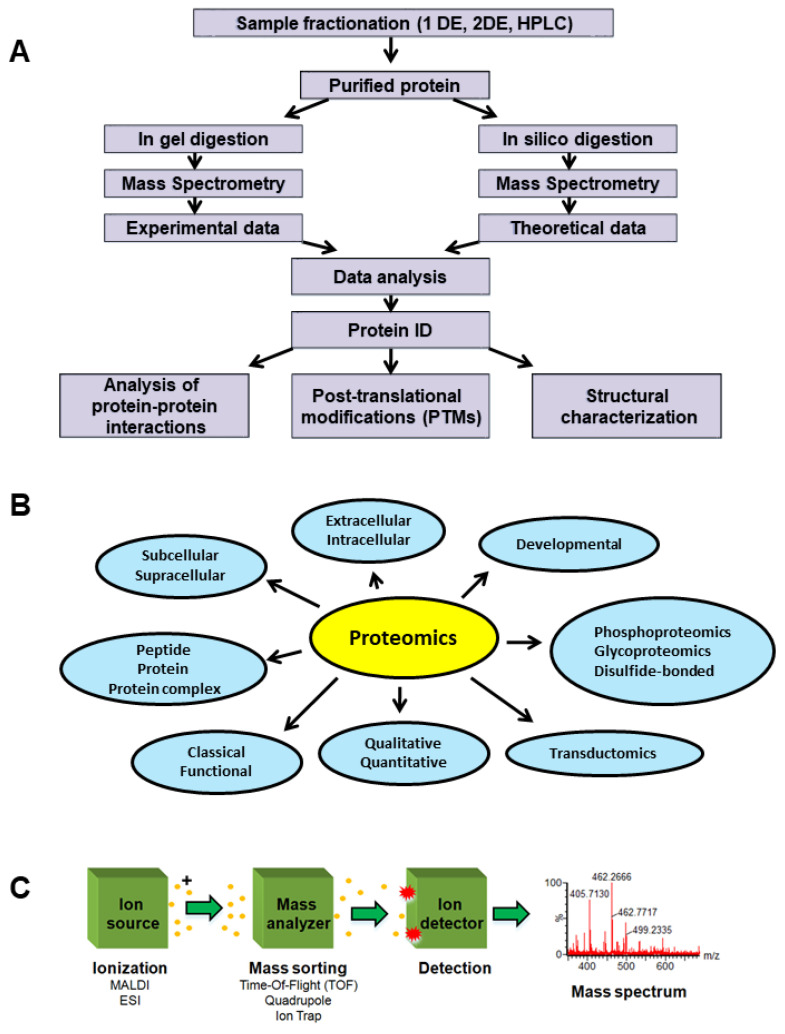
General proteomics experiment. (**A**) Proteomics experiment workflow schematic. (**B**) Proteomics and applications schematic. (**C**) Mass spectrometer schematic. Reprinted and adapted with permission from Sokolowska, I.; Woods, A.G.; Wagner, J.; Dorler, J.; Wormwood, K.; Thome, J.; Darie, C.C. Mass spectrometry for proteomics-based investigation of oxidative stress and heat shock proteins. In *Oxidative Stress: Diagnostics, Prevention, and Therapy*; Andreescu, S., Hepel, M., Eds.; American Chemical Society: Washington, DC, USA, 2011 [25]. Copyright @ 2011, American Chemical Society.

Co-immunoprecipitation techniques are useful for identification of protein interactomes/PPIs in BC cell extracts [9]. Western blotting (WB), immunohistochemistry (IHC) [10], enzyme-linked immunosorbent assays (ELISA) [11], forward-phase protein arrays [12], reverse-phase protein arrays (RPPA) [13,14], and MS-based methods are commonly used protein analysis for tissue and cell samples [13,15]. Undoubtedly, in diagnostic and even within research laboratories in oncological pathology, IHC-based methods are still widely used as morphology-based assays for in situ analysis of target proteins as specific tumor antigens [16]. IHC is able to highlight the biomolecular architecture at organ, tissue, cell, and subcellular level, and can be used to diagnose and classify into subtypes and assess the grade and treatment efficacy in various malignancies [17]. However, multiplex IHC based on fluorescence microscopy is generally limited to the simultaneous detection of 3–5 biomarkers, with hyperspectral/multispectral methods limited to eight [17]. Withal, most of the developed IHC assays are qualitative or semi-quantitative but not quantitative [18]. WB and IHC, are both antibody-based techniques traditionally used to assess the protein level, but are also used to detect protein isoforms [18]. In immunofluorescence (IF) microscopy, only one protein isoform may be commonly targeted at a time because the fluorescence spectral overlap [18]. This may occur when excitation and emission wavelength of one fluorophore includes the spectrum of the other used fluorophore [19]. Additionally, these techniques may lack specificity and reproducibility, while the comparison between the expression levels in simultaneous detection of many protein isoforms is available only when the antibody recognizes an identical epitope for the analyzed isoforms [18]. The simultaneous and accurate quantification of protein isoforms in biological samples may be performed by LC-MS/MS technique [20] as well as by MALDI-MS/MS-based proteomics [21]. The resulted values obtained by MS-based proteomics (Figure 1) may be compared for validation with those obtained using WB and IHC. Thus, a potential isoform-based diagnosis in BC successfully combines WB/RPPA/IHC/IF and MS-based proteomics techniques that are used within the same experimental design to detect and quantify the isoforms of different proteins, such as estrogen receptor (ER) [22] or folate receptor (FR) [18]. 

**Figure 2:**
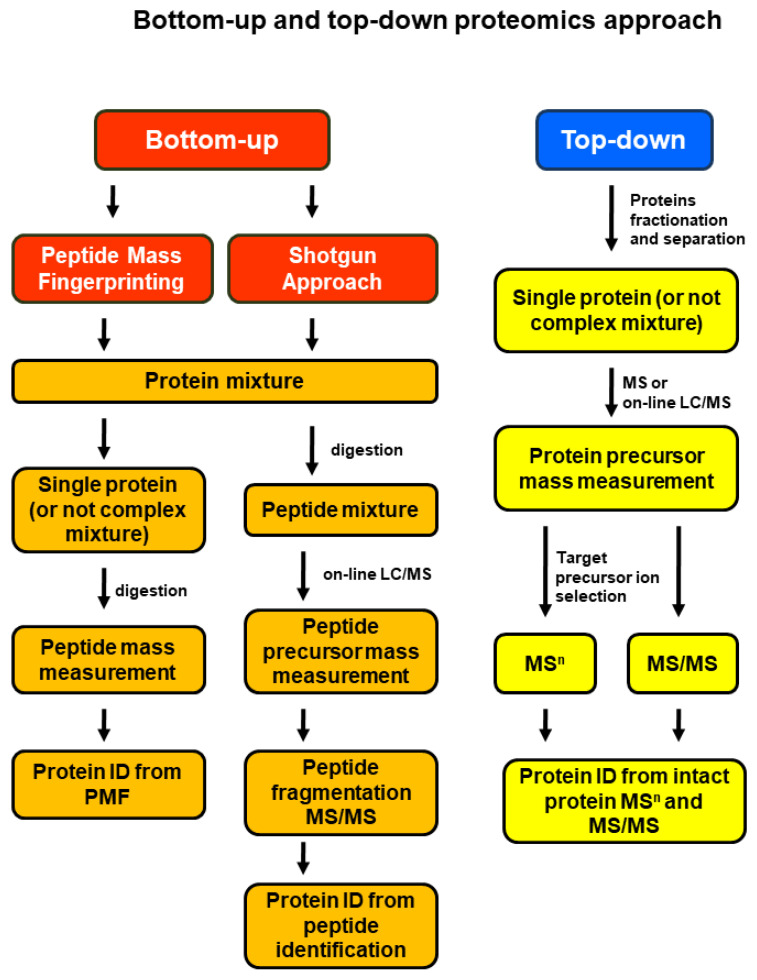
Schematic workflow for bottom-up and top-down MS-based protein characterization and identification. Reprinted and adapted with permission from Woods, A.G.; Sokolowska, I.; Ngounou Wetie, A.G.; Channaveerappa, D.; Dupree, E.J.; Jayathirtha, M.; Aslebagh, R.; Wormwood, K.L.; Darie, C.C. Mass Spectrometry for Proteomics-Based Investigation. *Adv. Exp. Med. Biol.* **2019**, *1140*, 1–26. [26]. Copyright @ 2019, Springer Nature Switzerland AG.

**Figure 3:**
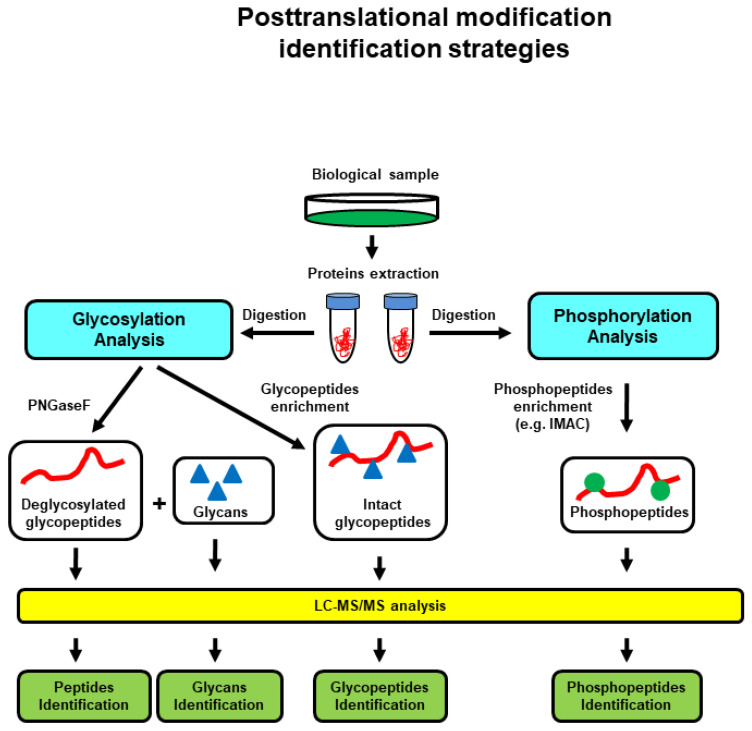
MS-based characterization of protein PTMs (glycosylation and phosphorylation), general strategies. Reprinted and adapted with permission from Woods, A.G.; Sokolowska, I.; Ngounou Wetie, A.G.; Channaveerappa, D.; Dupree, E.J.; Jayathirtha, M.; Aslebagh, R.; Wormwood, K.L.; Darie, C.C. Mass Spectrometry for Proteomics-Based Investigation. *Adv. Exp. Med. Biol.* **2019**, *1140*, 1–26. [26]. Copyright @ 2019, Springer Nature Switzerland AG.

**Figure 4:**
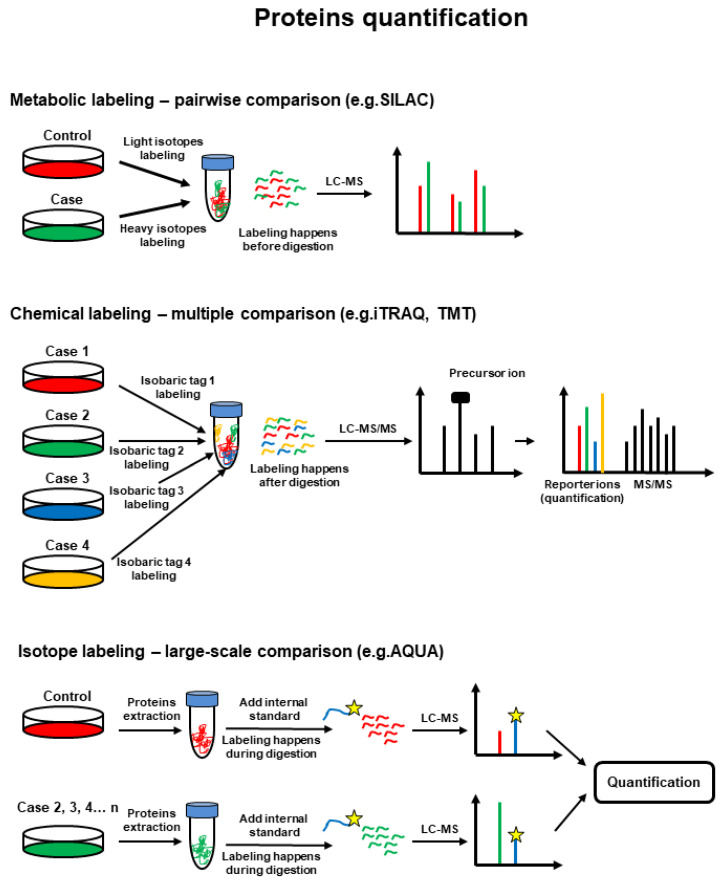
MS-based protein quantification workflow strategies via stable isotope labeling. Reprinted and adapted with permission from Woods, A.G.; Sokolowska, I.; Ngounou Wetie, A.G.; Channaveerappa, D.; Dupree, E.J.; Jayathirtha, M.; Aslebagh, R.; Wormwood, K.L.; Darie, C.C. Mass Spectrometry for Proteomics-Based Investigation. *Adv. Exp. Med. Biol.* **2019**, *1140*, 1–26. [26]. Copyright @ 2019, Springer Nature Switzerland AG.

There are two main approaches for MS-based proteomics (Figure 2). In bottom-up proteomics, the protein mixtures are digested and the resulting peptide mixtures are analyzed by liquid chromatography (LC)-MS and LC-tandem mass spectrometry (LC-MS/MS or shotgun approach) or separated by electrophoresis and then individual proteins are digested and analyzed by Matrix Assisted Laser Desorption/Ionization (MALDI)-MS in a method called peptide mass fingerprinting. In top-down proteomics, intact/whole proteins or a mixture of proteins are analyzed for molecular mass in MS mode and further fragmented to provide partial fragments in MS/MS mode. Thus, the target protein’s mass is identified and its amino acid sequence is confirmed by MS/MS fragmentation [23]. The top-down approach allows the analysis of PTMs at the intact protein level [24], while bottom-up proteomics can be used for the identification of a peptide, protein, PTM in a peptide/protein (Figure 3), and for quantitative proteomics [20] (Figure 4). 

However, in situ peptidomic and proteomic landscape is lost in analyses based on solid tissue sample homogenization. The analysis of tissue sections has become feasible due to MALDI mass spectrometry imaging (MALDI-MSI). In this manner, MS-based proteomics links specific clinical biomarkers to the rest of the proteome [27]. MSI facilitates a high level of multiplexing without the limitations of the optical methods [17], thus leading to the discovery of novel biomarkers [28]. Some relatively new methodological approaches, such as the targeted multiplex mass spectrometric imaging (TAMSIM), that is a matrix-free laser desorption/ionization (LDI) method, use antibodies conjugated to small organic photocleavable mass-tag that are cleaved and ionized during MSI [17]. A new method combining MALDI-MSI with IHC, termed MALDI-IHC, has been described, allowing the high-plex MSI of a wide range of biomarkers in various tissues, including BC [17]. In advanced HER2+ invasive ductal carcinoma, MALDI-MSI emphasized the association between intratumor heterogeneity and the prognosis of BC; the higher heterogeneity of tumors with a better prognosis reflects the presence of infiltrating immune cells that facilitate the treatment response [29]. An integrated experimental design based on IHC and histology-directed MSI defined the proteome profile of tumor microenvironment (TME), suggesting that the phosphatase and tensin homolog (PTEN) expression may be associated with different collagen types and regulation by PT sites of modification [30].

In clinical tissue-based proteomics following surgical procedures applied to solid tumors, fresh frozen tissues sections (FF), optimal cutting temperature embedded (OCT), and formalin fixed and paraffin embedded (FFPE) materials, that are sometimes affected by bio-banking limitations, may be analyzed with preservation of the tissue’s proteome dynamics [31]. Laser capture microdissection (LCM) allows different regions of the same tissue sample to be compared. In BC, proteomics of human and animal models bodily fluids is based on analysis of many clinical samples obtained in a minimally-invasive or non-invasive procedure, i.e., liquid biopsies such as blood/plasma/serum, urine [32,33,34], nipple aspirate fluid (NAF) [35], saliva [36,37], tear fluid [38,39,40], and milk [41,42]. Additionally, proteomic profiles and dysregulated proteins from various animal and cell-based model systems for BC have been analyzed to facilitate the discovery of novel biomarkers and to characterize proteins of interest, such as immortalized BC cell lines [43] grown in 2D and 3D conditions, organoid models [44], cell line-derived xenograft (CDX) or patient-derived xenograft (PDX) models, cell-free BC patient-derived scaffolds (PDSs), and animal models [45]. MS analysis of circulating tumor cells (CTCs), which are precursors of metastasis in cancer, led to the identification of abundant protein content dynamics in CTCs collected from xenograft models of BC, to emphasize the metastasis mechanisms [46]. 

First of all, MS is useful to identify BC subtypes at the protein level and to assess the accurate quantification of biomarkers, signaling pathways, and biological pathways [47]. In this context, MS-based omics, including proteomics-based investigations of dysregulated proteins, is essential for characterization of TNBC [48]. Surface-enhanced laser desorption/ionization time-of-flight (SELDI-TOF MS) protein chip technology was successfully applied to discover a unique combination of serum biomarkers for BC as an independent sample panel, while MALDI-TOF/TOF MS accurately identified these biomarkers in the panel [49]. MALDI-TOF MS demonstrated that the luminal, HER2+, and TNBC subtypes of BC have different protein and lipid profiles [50], while MALDI-MSI strongly discriminated between cancer and benign tissue in TNBC, based on the identification network of proteins that could be used as biomarkers of disease recurrence in patients with this BC subtype [28]. It was demonstrated that MALDI MS is easy to use, reproducible, and high-throughput technology, which provides a cheaper and faster alternative to genetic and IHC approaches [51]. LC-MS/MS analysis identified stage-specific protein expression profiles of BC tissues, considering the interaction, function, networks, signaling pathways, and protein pathways involved in each profile. This has application in the discovery of novel biomarkers in breast carcinogenesis [52]. High-resolution and high-accuracy MS identified high-expression levels of proteins associated with protein turnover in BC tissues that lead to metabolic alteration and remodeling of tissue homeostasis, as well as significant proteomic differences between BC stages and minor differences between primary tumors and lymph node metastases [53]. Quantitative LC-MS/MS methods are also useful in analysis of proteins involved in metastasis of BC for diagnosis, prognosis and understanding of metastatic mechanisms [54]. Liquid chromatography-MS shotgun analysis is also useful to identify new subtypes of BC, such as the TN-like ER+ [55] or to analyze the proteome of mammary organoids, emphasizing distinct signatures after exposure to environmental chemicals [44]. LC-MS/MS proteomic studies of BC cell lines, such as MCF7 treated with different therapeutic agents, revealed changes in protein expression profiles related to glycolysis, actin signaling, and energy metabolism [43]. NanoLC-MS/MS was used to investigate the molecular functions, biological processes and underlying mechanisms through which dysregulated proteins analyzed in MCF7 BC cell line in overexpressed [56] and downregulated jumping translocation breakpoint protein (JTB) condition cause increased cell growth, proliferation and invasion, as well as for potential use as a biomarker in breast cancer [57]. Proteomic profiling based on LC-MS/MS of the extracellular matrix (ECM) of xenograft BC metastases in different organs revealed distinct metastatic niches created by tumor and stromal cells in the brain, lungs, liver, and bone marrow, all derived from parental MDA-MB-231 TNBC cells, suggesting that some niche-specific ECM proteins could be involved in metastatic tropism [58].

Numerous studies in oncobreastomics compared immunohistochemical detection and proteomics technologies to the integrate available BC proteomic datasets and to validate and identify new prognostic biomarkers candidates [15]. The combination of MALDI-MSI, LC-MS/MS, and IHC provides a valuable tool for characterizing the molecular heterogeneity of tissues and identifying new biomarkers for a more personalized therapy [59]. Moreover, MS provides large-scale measurements of relative protein levels, changes to protein conformation and protein–protein interactions that occur upon cancer treatment [60] or quantifies changes in protein structure and interactions in multi-drug resistant human carcinoma cells [61]. In order to ensure a complete tumor removal during breast conserving surgery, a modern technology that couples a handheld and biocompatible MasSpec Pen to a mass spectrometer has been developed to differentiate the molecular patterns of normal breast tissue and lymph node from invasive cancer tissue [62] or to assure direct molecular analysis of in vivo and ex vivo freshly excised tissues in human surgery performed on different tumors, including BC [63]. In the following subchapters, several proteomics-based investigations of dysregulated proteins in BC tissue and liquid biopsies will be discussed, with reference to biological processes (BP), cellular components (CC), and molecular mechanisms (MM) that are involved in BC tumorigenesis and metastatic mechanisms.

## 2. Proteomics-Based Investigation of Dysregulated Steroid Receptors and HER2

Steroid receptors are a family of nuclear receptors that function as transcription factors (TFs) that mediate the mitogenic effects of estrogens [64]. This TF family includes estrogen receptors (ERs), progesterone receptors (PRs), and androgen receptors (ARs) that are usually assessed by IHC approaches. In BC, a 3-marker panel of ER, PR, and human epidermal growth factor receptor 2 (HER2/ErbB2) IHC staining has been frequently used [65] (Table 1). However, as a very heterogeneous collection of neoplastic diseases, BC requires a larger pool of candidate biomarkers beyond ER, PR, and HER2 proteins for diagnosis and personalized BC treatment [66]. Even though IHC provides good results, MALDI-TOF MS assures a large diagnostic potential, but, due to its relatively recent development and high cost, its use in clinical practice remains uncommon [67]. However, LC-selected reaction monitoring (LC-SRM) MS quantifies protein biomarkers across a wide range of expression levels from a single sample, providing multiplex analysis in BC tissue, such as ER, PR, and HER2, in interactions with other proteins, emphasizing key aspects of breast tumor biology [27]. 

Both ER subtype alpha (ERα), that functions as a promoter of cell proliferation in BC, and beta (ERβ) isoform, which suppresses cell proliferation [68], regulate transcription of different estrogen target genes [67]. ERs are expressed in many cells and tissues [69], but these receptors are critical in the development and progression of BC [70]. More than two thirds of all human BC are ER+, based on the detection of ER expression by IHC in at least 1% of the tumor cells [71], reflecting the importance of this protein for diagnosis and treatment strategy [67]. The first study to examine differences in proteomic expression among breast tumor versus normal mammary epithelium and ER+ versus ER- tumors, using MALDI MS coupled with LC-MS/MS and IHC analysis of tumor cells acquired by laser capture microdissection (LCM) from frozen tissues, has been published by Sanders et al. in 2008 [51]. Phosphorylation by different kinases on different sites of the ER is involved in tamoxifen resistance [72], while acetylation, ubiquitination, SUMOylation, and methylation have been also described as frequent PTMs of ERs [73]. A systematic mapping of PTMs of human ERα with emphasis on novel phosphorylation sites was reported using peptide mass fingerprinting by MALDI-TOF MS, peptide identification by tandem MS and nano-LC-multiple reaction monitoring (MRM) MS that occur in endogenous proteins isolated from the estradiol-stimulated MCF7 human BC cell line [70]. This experiment demonstrated the sensitivity of tandem MS methods for detection of phosphorylation sites in low level proteins such as ERα. 

HER2 is a transmembrane tyrosine kinase receptor glycoprotein from the epidermal growth factor family (EGF) that regulates cell growth, survival, differentiation, and proliferation [74]. HER2 is overexpressed in 15–20% of BC patients and it is an important biomarker of poor prognosis [75,76]. When overexpressed in BC it provides the cell with anti-apoptosis signals [76]. The hyper-activated HER2 and the autophosphorylation of tyrosine residues within the cytoplasmic domain of this receptor leads to uncontrolled cell growth, proliferation, and tumorigenesis [74]. MALDI MSI and protein identification performed by tissue microextraction and fractionation followed by top-down tandem MS have successfully assessed the HER2 status directly from BC tissues [77]. 

## 3. Proteomics-Based Investigation of Transcriptional and Translational Dysregulation in BC

Transcription and translation are frequently deregulated in cancer [78]. Dysregulated transcription and signaling pathways are associated with cancer development. The aberrant expression of RNA-binding proteins (RBPs) reveal their importance in the progression of many cancers [79]. In BC, overexpressed and downregulated RBPs are involved in RNA processing, splicing, localization and RNA silencing, DNA transposition regulation, methylation, alkylation, mitochondrial gene expression, transcription and translation regulation, estrogen response, and inflammatory mediators [80]. A complex protocol using RNA affinity purification followed by MS allows a profile of the RNA-sequence-interacting proteome and to identify RBPs of interest [81]. To define the BC cell invasion mechanisms and contributing pathways, two-dimensional gel protein electrophoresis (2D-PAGE) and MALDI-TOF were used in the analysis of FF invasive intra-ductal carcinoma samples coupled with IHC analysis of FFPE malignant and non-malignant BC specimens. Thus, the upregulation of calreticulin (CRT) expression in tumor tissues compared to the normal adjacent tissues indicated that CRT mediates invasive cancer characteristics through the transcriptional dysregulation of p53 and MAPK pathways [82]. Cancer cells break the mechanisms that govern translational regulation in protein synthesis, at level of initiation, elongation, termination, and recycling. Multiple oncogenes and signaling pathways are activated, upregulated or mutated; consequently, translational dysregulation allows cancer cells to adapt to a hostile TME [83]. The core binding factor subunit beta (CBFB) is a transcription factor involved in translation regulation in cytoplasm and transcription regulation in the nucleus, with the CBFB gene being frequently mutated in several solid tumors, including BC [78]. A complex experimental design that includes IHC/IF and mass spectrometry analysis showed that CBFB suppresses BC through orchestrating translation and transcription [78]. Other deregulated proteins involved in transcriptional and translational dysregulation and detected by proteomics techniques are listed in Table 1.

## 4. Proteomics-Based Identification of Dysregulated Proteins Involved in BC EMT, Invasion and Metastasis

Tumor metastasis formation at distant sites includes several steps, such as breaching of basement membrane, escaping from the primary tumor, migration to blood and lymphatic vessels, extravasation and movement into distant organs [84]. Epithelial-to-mesenchymal transition (EMT) is a complex process that induces molecular changes inside tumor cells and into their TME, resulting in loss of epithelial biomarkers and acquisition of mesenchymal characteristics, which promotes the invasive and migratory cellular phenotype [85]. Proteomics approaches are essential to increase the understanding of the complex molecular mechanisms of EMT at the protein level by analysis of proteomic alterations (Table 1).

LC-MS/MS analysis applied to MCF7 and MDA-MB-231 breast cancer cell lines showed that the TFs overexpression, such as SNAIL, SLUG, ZEB1/2, and TWIST1, induces EMT in correlation with cancer aggressiveness [85]. 

The EMT process is closely linked to the alteration of intracellular cytoskeleton and ECM remodeling to facilitate local invasion in cancer [86]. Intermediate filament (IF) proteins, such as cytokeratins (CK), vimentin (VIM), the most abundant IF protein [87], and neuroepithelial stem cell protein (nestin/NES), are the largest families of cytoskeletal proteins assuring the structural integrity in cells and tissues, serving as diagnostic biomarkers in cancer cells that usually emphasize characteristic alterations in IF gene expression and protein regulation [88]. VIM, a key protein involved in EMT, was detected as overexpressed in BC cells, especially in basal-like BC (BLBC) subtype [89]. Co-immunoprecipitation-MS analysis revealed that p62, a signaling adaptor frequently overexpressed in cancer and functioning as a tumor metastasis promoter, positively interacts with VIM [90]. Thus, a proteomics experiment demonstrated that VIM mediates the function of p62 in BC invasion [90]. Moreover, MALDI-TOF MS/MS is useful to characterize the interaction mechanisms between VIM and several derived phytochemicals, such as vinyl disulfide-sulfoxide ajoene from garlic, that results in the disruption of the VIM filament network and induces anti-metastatic activity in MDA-MB-231 BC cell line [91]. ER+ BC often contain subpopulation of cells that express the intermediate filament protein cytokeratin 5 (CK5) [92]. Immunoprecipitation and MS has been performed to emphasize CK5 interacting proteins in ER+ BC cells, identifying that the blockade of CK5-β-catenin interaction may reverse the detrimental proprieties of CK5+ breast cancer cells [92]. The most abundant studied PTM in IFs is phosphorylation that is involved in regulation of IFs dynamics, modifying the protein itself and creating binding sites for other proteins [87]. VIM IFs become weaker with increasing amounts of phosphorylated protein, adapting cells to specific TME conditions [87]. MALDI-TOF MS is able to quantify VIM phosphorylation in BC cells, predicting poor overall survival (OS) or metastatic disease, representing a new prognostic biomarker for BC patients [89]. 

Several ECM components also trigger the EMT process [85]. Fibrillar collagen types I and III are dominant in the extracellular matrix (ECM) [93] and are involved in attachment of cells to ECM molecules, either directly or via extracellular collagen-binding proteins, all of which are involved in cancer cell adhesion and migration [94]. A considerable degradation of ECM components, including collagen molecules, are required for cancer cell locomotion [95]. An extensive deposition of fibrillary collagen in the TME promotes cancer progression and metastasis, followed by low survival rates for patients [96]. LC-MALDI TOF/TOF MS analysis, corroborated with iTRAQ data for 22 isoforms of collagen type I alpha-1 chain (COL1A1), indicated an increase in fibrillary collagens in invasive ductal carcinoma (IDC) compared with little change in expression in fibroadenoma (FA) or ductal carcinoma in situ (DCIS) [97]. COL1A1 upregulation was associated with metastases and poor survival, especially in patients with ER + BC [98]. LC-MALDI-MS/MS and MALDI-TOF MSI analyses showed the upregulation of COL1A1 and COL1A2 in invasive breast cancer and COL6A3 in almost all breast cancer samples [28]. Collagen type III alpha-1 chain (COL3A1) functions in cell adhesion, migration, proliferation and differentiation by its interations with collagen-binding integrins, which are transmembrane receptors mediating cell adhesion [99] and breast cancer development [100]. Within a complex proteomic experimental design, LC-MS/MS analysis showed that integrin ITGB3-mediated uptake of small extracellular vesicles (EVs) facilitates intercellular communication in BC cells [101]. COL3A1 upregulation was positively related to a worse prognosis, advanced tumor stage, local recurence and invasion [102], tumor-infiltrating immune cells (TIICs) recruitment, ECM-receptor interaction, and regulation of actin cytoskeleton and adhesion pathways [103]. COL3A1 was overexpressed in lymph nodes affected by metastatic ductal breast carcinoma cells [104], and also in DCIS myoepithelial cells compared with normal mammary myoepithelium [105]. LC-MS/MS analysis also identified collagen type V in stage 2 of BC, emphasizing its role in tumor progression [52], and quantified type XIV collagen as a prognostic factor and diagnosis biomarker differentially highly expressed in metastatic tissues of patients with massive lymph node involvement compared with non-metastatic tissues [54]. A recently reported method called ECM imaging mass spectrometry (ECM IMS) has been used to analyze the stromal proteins of BC progression, including the dysregulated collagen type patterns in FFPE tissue biopsies [106]. Previously, MALDI MSI analysis identified proteomic differences in BC-associated stroma for identifying biomarkers of stromal activation in BC [107]. To identify and quantify the functional tumor-stroma inter-relationships between tumor cells and cancer-associated fibroblasts (CAFs), a complex approach based on MALDI MSI and LC-MS/MS detected high levels of collagen 1 (COL1A) and alpha smooth muscle actin (α-SMA) in ER-negative BC patients with value of prognostic factors for cancer progression [108].

## 5. Proteomics-Based Identification of Dysregulated Proteins Involved in Intermediary Metabolism Reprogramming in BC Cells

One of the most important features of cancer cells compared to healthy cells is metabolic reprogramming or altered metabolism [109]. Aerobic glycolysis, the main metabolic pathway in tumor cells, is also involved in EMT process, with consequences in tumor progression [110]. BC cells emphasize high expression of glucose metabolism-related enzymes and glucose transporters (GLUT) [111]. Using a LC-MS/MS protocol, pyruvate kinase M (PKM), an enzyme involved in HALLMARK_GLYCOLYSIS, metabolic reprogramming, cancer cell proliferation, adaptation to oxidative stress-induced apoptosis [112] and UDP-glucose 6-dehydrogenase (UGDH), involved in hyaluronic acid production and BC progression [113], have been found to be upregulated into a MCF7 BC cell line transfected for downregulation of jumping translocation breakpoint (JTB) protein [57]. 

Regulation of lipid metabolism in cancer cells under metabolic stress is related to cell membrane biogenesis, energy production and protein modification [114]. Two-dimensional gel electrophoresis (2-DE) and two-dimensional fluorescence difference gel electrophoresis (2D-DIGE) coupled to MALDI-TOF/TOF are useful to explore BC metabolism at the proteome level by detection of changes that occur in triacylglyceride (TAG) metabolism and metabolism-associated proteins, such as glycerol-3-phosphate dehydrogenase 1 (GPD1) and monoacylglycerol lipase (MAGL) that were found as downregulated in tumor breast tissue compared to healthy tissues [115]. Other dysregulated enzymes involved in cancer cell metabolism reprogramming and detected by proteomics technics are listed in Table 1.

**Table 1:** Proteomics-based investigation of dysregulated protein involved in BC.

Protein	Gene Name	Biological and Pathological Role in BC	Methods of Identification	Status in BC	Potential Clinical Use
**Steroid receptors and HER2**					
Estrogen receptors	ER isoforms: ERα & ERβ	Nuclear receptors/TFs that regulates transcription of estrogen target genes [67]; ERα is a promoter of cell proliferation/tumorigenesis in BC, and ERβ suppresses cell proliferation [68]	IHC [116], MALDI-TOF MS [67], LC-SRM MS [27]; multiplex IHC-MALDI-MSI (MALDI-IHC) [17]	More than 70% of all BC are ERα [117]	Diagnostic biomarkers, classification of BC subtypes [67]
			nLC/ESI-MS/MS; MALDI-MS/MS (MS^n^) [70]	PTMs and PPI modulate activity: ubiquitination [117]; phosphorylation [70]	Tamoxifen resistance [72]
Progesterone receptors	PR isoforms: PRA & PRB	TFs that modulate ERα action in BC [118]; exhibits both activatory and repressive effect on gene transcription [119]	IHC [116]; LC-SRM MS [27]; multiplex IHC-MALDI-MSI (MALDI-IHC) [17]	Association between ERα/PR induces cell proliferation and tumor growth [120]	Predictive biomarker [121], prognostic and predictive biomarker of response to endocrine therapy [118]
Androgen receptor	AR	Nuclear TF that mediates the biological effects of androgens; tumor suppressor in ER+ BC and inducer of tumor progression in ER- BC, including HER2+ and TNBC [68], it has a crucial role in BC pathology and progression [122]	IHC [123], PRM targeted proteomic [124]	Expressed in 70–90% of the BCs [122]; upregulated in luminal A & B subtypes of BC and a subset of TNBC; positive immunostaining was associated with smaller tumor size [123]	Possible prognostic biomarker [123]; potential therapeutic target in AR+ BC patients [122]
Human epidermal growth factor receptor 2	HER2/neu, c-erbB2	Membrane tyrosine kinase and oncogene [76]; regulates cell growth, survival, differentiation and proliferation [74]	IHC, FISH, CISH, SISH [76]; MALDI-MSI [77], LC-MS/MS+SRM assay+FISH+IHC [125]; LC-SRM MS [27]; multiplex IHC-MALDI-MSI (MALDI-IHC) [17]	Overexpressed in 20–30% of BC [76]	Predictive and prognostic biomarker; treatment target [76]; poor prognosis and increased likehood of metastasis especially in node-positive BC [126]
**Transcription and translation regulation**					
Core binding factor subunit beta	CBFB	Translation regulation in cytoplasm and transcription regulation in the nucleus [78]	IHC, IF, immunoblotting, MS [78]	Highly mutated in solid tumors, including BC [78], mutations mainly occur in HR+/HER2- BC [127]	Putative prognostic biomarker in HR+/HER2- BC [127]
Catenin beta 1	CTNNB1	Transcriptional regulation in the Wnt signaling pathway and cell adhesion molecule by linking cadherins to the actin cytoskeleton [92]; downregulation inhibited cell proliferation, migration, and invasion and induced apoptosis in RCC [128]	LC-MS/MS [56]; IHC [129]	Key role in most cancers as an oncogene [128]; β-catenin/Wnt pathway activation is preferentially found in TN-BL breast carcinomas [130]	Prognostic biomarker [131]; poor clinical outcome in BC [130]
Histone H1	H1 (seven somatic proteoforms [132])	Chromatin organization and transcriptional regulation; knock-down in BC results in altered gene expression, proliferation, and IFN response [133]	Immunoblotting, IHC, LC-MS, LC-MS/MS [132]	H1 showed PTMs in BC cells [133]	Putative biomarker of proliferation BC cells [132]
**EMT, cytoskeleton reorganization, cell adhesion, ECM, invasion and metastasis**					
Vimentin	VIM	EMT; intermediate filament family protein; in IDC is associated with low ER, low PR, increased basement membrane invasiveness, and resistance to BC chemotherapy [134]	IHC [134], IF [135]; LC-MS for detection of phosphorylated isoform that increases mobility in cancer cells [87]; MALDI-TOF MS/MS for detection of methylated isoform [89] and interaction VIM-garlic phytochemical with anti-metastatic activity [91]	Overexpressed in BC, especially in BLBC [89]	Mesenchymal marker, poor prognostic factor of BC [134]
Epithelial (E)-cadherin	CDH1	EMT; adhesion molecule of the epithelial adherens junction; dual role in BC: putative tumor suppressor [136] or promotor of metastasis and invasiveness [137]	IHC [137,138], IF [135]; 2D-DIGE and MS [139]	Downregulated in BC [140]	Phenotypic marker; biomarker of tumor subtypes [136]; prognostic biomarker for patients with lymph node metastasis and TNBC [141]
Filamin A	FLNA	EMT; actin cross-linking protein, involved in regulation of BRCA1 expression in BC [142]	IHC [142]; LC-MS/MS [56]	Upregulated in BC, especially in myoepithelial cells [142]	Putative prognostic biomarker [142]
Pleckstrin homology domain-containing family G member 2	PLEKHG2	Actin cytoskeleton reorganization and transcriptional regulation, regulation of cell morphology [28]	MALDI-MSI, LC-MALDI-MS/MS [28]	Phosphorylated in TNBC [28]	Prognostic biomarker [28]
SRY-related high-mobility-group (HMG) box 11	SOX11	Transcription factor and embryonic mammary epithelial marker associated with mesenchymal state and embryonic phenotype of BC cells [135]; involved in BC growth, migration, and invasion, regulating the BLBCs phenotype [28]	WB, IF [135]; IHC [143], MALDI-MSI, LC-MS/MS [28]	Upregulated in BLBC [144]	Prognostic biomarker [28] for BC with elevated risk of distant metastases and poor outcome [135], therapeutic target [28]; ER negative DCIS SOX11+ tumor cells metastasize to brain and bone at greater frequency than in lungs [135]
Collagen type I alpha 1 chain	COL1A1	EMT; promotes BC metastasis [98]; upregulation is a risk factor for radiation-associated secondary diseases in BC [145]	IHC [98], MALDI-MSI, LC-MALDI-MS/MS [28]	Upregulated in invasive BC (IDC) [[28,97]	Prognostic biomarker [28], poor survival in ER+ BC, potential therapeutic target [98]
Collagen type I alpha 2 chain	COL1A2	EMT; ECM assembly; upregulation is a risk factor for radiation-associated secondary diseases in BC [145]	MALDI-MSI, LC-MALDI-MS/MS [28]	Upregulated in invasive BC [28]	Prognostic biomarker [28]
Cytokeratins	CKs	IFs [146]; CK+ cells are enriched in cancer stem cell proprieties [92]	IHC [146]; multiplex IHC-MALDI-MSI (MALDI-IHC) [17]	CK 5/6 upregulated in ER+ BC and BLBC [147]	Adjuncts in diagnosis, classification and prognostication of BC [146]
**Intermediary metabolism reprogramming**					
Fatty acid synthase	FASN	FAM; enhances malignant progression [148], migration, metastasis [149], proliferation, drug resistance, and apoptosis [150]; inhibition reduces cell proliferation, suppresses migration and invasion and induces apoptosis [151]	LC-MS/MS [56], MALDI-TOF/TOF MS/MS [126]; IHC [150]	Overexpressed in cancer cells [148]; highly expressed in different sex hormone-related malignant tumors, positive expression in TNBC correlated with lymph node metastasis and stage [150]	Prognostic biomarker in TNBC [150]
Triose-phosphate isomerase	TPI1	Glycolysis; promotes tumor development and progression of BC in tissue and cell lines, proliferation, metastasis, activates PI3K/Akt/mTOR, regulates EMT [152]	WB, IHC, IF [152]; MALDI-TOF/TOF MS/MS [126]	Upregulated in multiple cancers [152]	Therapeutic target for BC [152]
Alpha-enolase	ENO1	Cell growth, hypoxia tolerance, autoimmune activities, glycolysis pathway [153]	WB [154], IHC [155], LC-MS/MS [156]; MALDI-TOF/TOF MS/MS [126]	Upregulated in BC [153,154]	Prognostic biomarker [155,157]
Phosphoglycerate kinase 1	PGK1	Glycolysis, hypoxia; cancer progression, metastases; invasion promoter, regulates HIF-1α-mediated EMT [158]	MALDI-TOF/TOF MS/MS [126]	Overexpressed in BC [158]	Poor prognosis, potential survival biomarker in BC [158]
**Cell cycle, cellular division, mitotic spindle, cell proliferation**					
Jumping translocation breakpoint protein/prostate androgen regulated protein	JTB/PAR	Dual role: tumor suppressor or oncogene; involved in cell proliferation, tumorigenesis, genomic instability [159]	WB, immunoprecipitation, IF [159]	Overexpressed in many cancers, including BC [159]	Putative target for therapeutic intervention [159]
Beta-tubulin	TUBB	Carcinogenesis, metastasis [160]	LC-MS/MS [56]	Upregulated in BC tissue [160]	Potential prognostic biomarker for worse prognosis in ERα+ and better prognosis in ERα- BC [160]
Proliferation marker protein Ki-67	MKI67	Proliferation-associated nuclear antigen involved in cell proliferation and growth, migration, invasion, tumor progression, maintenance of stem cell characteristics [161]	IHC [162,163]; LC-MS/MS [56]	Overexpressed in cancer cells [164]	Marker of cell proliferation, prognostic and predictive biomarker in invasive BC [165,166]
Aminoimidazole-4- carboxamide ribonucleotide	ATIC	Cell proliferation [28]	MALDI MSI [28]	Upregulated in TNBC [28]	Putative prognostic biomarker [28] and therapeutic target in BC resistant to tamoxifen [28,167]
Mutant tumor suppressor p53 protein	TP53/mtp53	Driver oncogene [168], transcription factor involved in cell cycle; mtp53-related proteome targets cholesterol biosynthesis, DNA replication and repair pathways [168]	IHC [169,170], SILAC coupled to MS/MS [168]	The most frequently mutated gene in invasive BC; mutated in 30–35% of all BCs, and 80% in TNBC [171]	Potential biomarker and therapeutic target for BC patients, especially for TNBC [171]

BLBC-basal-like breast cancer; CISH-chromogenic in situ hybridization; ECM-extracellular matrix; EMT-epithelial-to-mesenchymal transition; FAM-fatty acid metabolism; FISH-fluorescence in situ hybridization; IDC-infiltrating ductal carcinoma; IF-immunofluorescence; IFN-interferon; IFs-intermediate filaments; IHC-immunohistochemistry; LC-SRM MS-liquid chromatography-selected reaction monitoring mass spectrometry; PRM-parallel reaction monitoring; RCC-renal cell carcinoma; SISH-silver enhanced in situ hybridization; TF-transcription factor; WB-Western blot.

## 6. Proteomics-Based Investigation of PTMs and PPIs in BC

The dynamics of PTMs in BC, which include phosphorylation, acetylation, glycosylation, methylation, oxidation, and ubiquitination [172], small ubiquitin-related modifier (SUMO)ylation, citrullination, and palmitoylation [173], alter protein localization, stability, and function [174], contributing to dysregulate cellular proliferation, adhesion and cell morphology [172]. Aberrant phospho-signaling is well known as a hallmark of cancer, MS being widely involved in identification of tens of thousands of phosphorylation sites [175]. The enrichment for phosphopeptides followed by reverse-phase liquid chromatography combined with LC-MS/MS is the most applied tool to decipher the phosphoproteome [176]. It is also known that the aberrant glycosylation is linked with BC development and progression [177], as well as the acetylation that promotes BC metastasis [173]. LC-MS/MS analysis aids the study of protein ubiquitination and could be used to discover novel biomarkers that are associated with BC progression [178]. Other frequently used proteomics approaches for PTMs analysis may be MALDI-TOF MS, ESI-MS/MS, and SELDI-MS [172].

LC-MS/MS analysis, alone or coupled with other MS techniques, is really useful to examine the PTMs profiles and expression patterns of modified proteins, leading to establishing of their potential use as biomarkers in BC: phosphorylation of histone H1 [132], nuclear factor of kappa light polypeptide gene enhancer in B-cells inhibitor (IκBα) [179], myeloid zinc finger 1 (MZF1) [180], α-isoform of the estrogen receptor (ERα) nuclear transcription factor in MCF7 BC cell lines identified by HPLC-ESI and MALDI MS [70], YWHAH adapter protein [181], cAMP-dependent protein kinase (PKA) [182], ACAP4, an ARF6 GTPase-activating protein [183], focal adhesion kinase (FAK2) [184], cytoskeleton proteins, such as cortactin (CTTN) as an actin-binding protein [185], γ-tubulin (TUBG1) [186]; acetylation of key nuclear proteins [187], ACAP4 [183]; N-glycosylation of membrane proteins [188]; ubiquitination and glycosylation of programmed death ligand-1 (PD-L1) [189]. 

MS-based techniques are able to detect the PTMs at the level of all cellular components, such as plasma membrane [188], cytosol [185], cytoskeletal microtubules [186], nucleus (nuclear transcription factors [179], nuclear protein kinases [181], histones [187]) or secreted extracellular vesicles (EVs) [190]. Proteomics techniques are essential to understanding the involvement of PTMs in the main mechanisms of biological processes and to decipher their molecular functions (i.e., ketohexokinase-A (KHK-A) signaling pathway that mediates fructose-induced metastasis in BC by YWHAH phosphorylation, which triggers cancer cell migration [181]; G protein-coupled estrogen receptor-1 (GPER)-induced signaling via GPER-mediated cyclic AMP-dependent protein kinase A (PKA)/BAD phosphorylation that is essential for the survival of BC stem cells (BCSCs) [182]; actin cytoskeleton signaling pathway involved in BC cell migration and invasion [185], pathways of mitotic spindle assembly [186], adaptation to changes in tumor microenvironment (TME) [183], promotion of the migratory activity of some cancer cell lines [183], signaling pathways activated in tamoxifen resistant BC cells like focal adhesion pathway [191]; and EMT-ome associated pathways [192]). Consequently, PTMs usually detected by LC-MS/MS and other associated proteomics techniques are important for modulation of protein–protein interactions (PPIs) [189], proliferation of malignant breast epithelial cells [182], tumor-associated immune escape [189], transcriptional activity [179,180], DNA damage response [187], apoptosis and necroptosis in breast cancer cells [179], metabolic reprogramming and the effect of nutrition on BC metastasis [181], cancer cell migration and metastasis [181], regulation of membrane trafficking, cytoskeleton remodeling and actin-containing stress fiber formation in migrating cells [183] or induction of the epithelial-mesenchymal transition (EMT) [185], the E-cadherin to N-cadherin switching considered as a molecular hallmark of EMT [180], and the abnormal morphology and compromised spindle function during mitosis and uneven chromosome segregation [186]. The interactome mapping by high-throughput quantitative proteomics analysis (IMAHP) performed by LC-MS^2^/MS^3^ technology was applied to a panel of 41 BC cell lines, emphasizing aberrant interactions that could serve as biomarker, predicting the drug sensitivity of cell lines [193]. 

A large portion of biochemical/biomolecular diversity in cells arises at the protein level and biomedical relevant proteoforms structures were identified during the last decades. Different biological processes, such as amino acid variation, alternative RNA splicing, post-translation modification (PTM), and post-translational cleavage, result in the formation of proteoforms. A proteoform family is made up of protein isoforms that are produced by the same gene however their biological features and function can vary substantially [194,195,196,197]. 

Each protein isoform has a unique molecular mass (Mr) and isoelectric point (pI) value as well as a different abundance levels in the cells. The first efficient techniques to separate those several isoforms of each protein were 2D gel electrophoresis and Western blot (2D-PAGE paired with corresponding protein specific antibodies) [198]. Furthermore, each PTM and splicing variant is carefully characterized using MS, particularly tandem MS/MS [194,195,197,198]. Several of the most studied PTMs in BC are listed in Table 2. 

## 7. Proteomics-Based Investigation of Dysregulated Proteins in Diverse Liquid Biopsies/Body Fluids

1. Blood-based proteomics

Either as blood proteins produced by the host immune system or as proteins secreted by tumors as the cancer secretome, serum or plasma circulating proteins play a key role in the development and progression of BC and represent an important source of biomarkers for determination of cancer risk, early diagnosis, treatment assessment, prognostication, and tumor progression monitoring [202]. Thus, protein biomarkers differentially expressed in blood can be used to establish non-invasive and tumor-specific blood-based tests for BC monitoring [203]. A unique combination of serum biomarkers for BC and the confirmation of this panel of biomarkers as an independent sample set has been performed by SELDI-TOF MS technique, while MALDI-TOF/TOF MS analysis was useful for the identification of these biomarkers, such as apolipoproteins (APOH, APOCI, APOI), C3a-desArg, and transthyretin (TTR) [49]. 2DE and MALDI-TOF MS were employed to detect differences in serum protein expression between patients with male BC (MBC) and healthy controls, emphasizing proteins involved in mitochondrial function (i.e., mitochondrial aldehyde dehydrogenase (ALDH2)), cell cycle regulation (cell division cycle 7-related protein kinase (CDC7)), lipid metabolism and transport (apolipoproteins APOA1 and APOE), apoptosis and immune response (clusterin (CLUS), CD5L, and CCL14), transcription (STAT3 and SSX3), invasion and metastasis, estrogen synthesis, and other biological processes [204]. In plasma samples of BC patients, nano-LC-MS/MS analysis identified several proteins differentially expressed as blood protein biomarkers for each stage of BC, associated with cell growth, ECM and cell-to-cell communication, energy metabolism and gene transcription, cell death and cancer development, transcription regulation, tumorigenesis and invasion, redox balance, and EMT [205].

Aberrantly externalized proteins produced by BC cells and stromal cells (i.e., by the mammary fat proteome [206]) accumulate in the tumor interstitial fluid (TIF) as a part of the TME, which can pass to the circulatory system. High-throughput LC-MS/MS profiling of the protein expression in TIF samples identified a panel of proteins as novel putative biomarkers associated with BC tumor status and subtype [203]. MALDI TOF/TOF-MS was used to analyze the secretory proteins of breast cancer-associated fibroblasts (CAFs) and normal breast fibroblasts (NFs), emphasizing that CAFs produce less collagens and matrix-degrading enzymes compared with NFs [207]. 

In BC, proteomic analysis of blood-circulating extracellular vesicles (EVs), such as exosomes/intraluminal vesicles (ILVs), microvesicles (MVs) and apoptotic bodies, is useful for early detection and diagnosis [208] due to their ability to function as carriers of transmembrane and non-membrane protein biomarkers in ECM and body fluids [209]. Exosomes and their molecular content play roles in development of BC, promoting tumorigenesis, metastasis, angiogenesis, immune escape, and treatment resistance [210]. Tandem-Mass-Tag (TMT)-based quantitative proteomics (LC-MS/MS) approach characterized the proteomes of individual patient-derived serum exosomes, identifying TNBC-derived exosomal proteins, including tetraspanin CD151 that promotes TNBC cell migration and invasion [211]. TMT labeling and nano-ESI-LC-MS/MS analyzed the entire exosomal cargo proteins as a potential multi-protein marker useful in BC diagnosis and monitoring of disease progression [212]. LC-MS/MS analysis of plasma EVs identified phosphoproteins significantly overexpressed in BC patients compared with healthy controls [213].

2. Proximal fluid proteomics/nipple aspirate fluid (NAF)-based proteomics has been performed by MALDI-TOF MS [214], LC-MS/MS [66] and SELDI-TOF MS [215] to identify patterns of proteins as proteomic signature for early BC detection. The proximal fluid, derived from the extracellular milieu of tissues, contains secreted proteins (secretome) at higher concentration than corresponding blood levels, thus providing a rich source for biomarkers discovery in BC [216]. A paired-proteomic shotgun strategy that relies on NAF analysis from both breasts of women with unilateral BC emphasized differentially abundant proteins involved in glycolysis and immune system activation, while the most abundant proteins confirmed a proliferative TME, particularly in ER+ BC samples [217]. A 2D-LC MS/MS-based study of NAF identified unique proteins, including BC associated biomarkers with origin in basement membrane, extracellular milieu and interstitial fluid surrounding breast cells that are involved in tissue homeostasis, cell-adhesion, and cell-to-cell communication, in correlation with stromal disruption and degradation, cancer cell proliferation, and migration [218]. nLC-ESI-Q-TOF MS technique emphasized that dried NAF spots on Guthrie cards analysis has putative applications for early BC screening and subtype classification [219].

3. Milk-based proteomics. Proteomics analysis of breast milk may identify biomarkers of BC risk [220]. nanoLC-MS/MS analysis emphasized the entire protein pattern in human milk samples from breastfeeding mothers with BC, who were diagnosed either before or after milk donation compared with healthy women emphasizing a wide panel of dysregulated proteins that may be considered as putative biomarkers for BC [42]. LC-MS/MS analysis quantified αS1-casein protein in human milk [221] that functions as a tumor suppressor through upregulation and hyperactivation of signal transducer and activator of transcription 1 (STAT1) signaling [222]. Proteomic analysis reveals induction of senescence and EMT in primary tumor and acceleration of cancer metastasis upon treatment with milk-derived extracellular vesicles (EVs) [223]. LC-MS/MS proteomic analysis was performed on EVs from breast milk that are loaded with active regulatory and stimulatory molecules with therapeutic potential [224], with identification of proteins involved in regulation of cell growth and inflammatory signaling pathways [225].

4. Urine-based proteomics. Urine is a useful, sensitive, non-invasive, and easy of sampling source of biomarkers, with a great potential for clinical use in the detection of BC. A LC-MS/MS proteomic approach led to the detection of upregulated proteins that may be used to identify pre-invasive BC in DCIS samples, early invasive and metastatic BC [32]. MALDI-TOF/TOF MS coupled with LC-MS/MS analysis identified urinary proteome alterations in HER2 enriched BC [33]. LC-MS/MS technique was useful to identify urinary proteome progressive changes during cancer development from a tumor rat model injected with Walker 256 breast carcinoma cells [34] commonly used to induce secondary brain tumors [226]. An optimization of urine sample preparation method for shotgun proteomics has been published [227].

5. Tear-based proteomics approaches generated protein biomarker profiles in tear fluid for BC patients compared to healthy women, using SELDI-TOF MS [40], MALDI-TOF/TOF MS [228], and LC-MS/MS techniques [39]. The upregulated or downregulated proteins are involved in ECM remodeling [39], host immune system pathways, and metabolic regulation [38]. 

6. Salivaomics/saliva-based diagnostics in BC. Saliva is a complex non-proximal fluid, its proteome reflecting both local and systemic disease [229]. Almost 27% of the whole-saliva proteins have been also identified in plasma [230]. Several MS-based approaches have been applied to detect salivary peptides, such as SELDI-TOF MS [231], [232], ESI-TOF MS, MALDI-TOF MS [233], ESI-Orbitrap MS, and ESI-Q-TOF MS [234]. The top-down analysis of undigested proteins has usually been performed using MALDI mass spectrometers, while the majority of digested proteins/peptides detections was performed using ESI ionization with TOF and Orbitrap mass analyzers [234]. A review published in 2017 showed that salivary biomarkers identified advanced stages of BC better than early stages, suggesting that a panel of biomarkers has a better ability to predict BC than individual biomarkers [235]. A meta-analysis and systematic review published recently assessed the accuracy of the diagnostic value of salivary biomarkers in differentiating between BC patients and healthy controls [236]. MS experiments led to a considerable protein list from whole saliva to create a more comprehensive catalog of human salivary proteins [237] and a catalogue of salivary proteins that are altered secondary to carcinoma of the breast [238]. LC-MS/MS was used to identify BC related salivary proteins that are modulated secondary to ductal carcinoma in situ (DCIS) of the breast [37] as well as for detection of overexpressed and downregulated proteins from saliva of patients with either HER2/*neu* positive or negative [36]. nLC-Q-TOF technology evaluated the proteomic profile of saliva and plasma from women with impalpable breast lesions. The changes in immune landscape, molecular transport and signaling pathways have been emphasized by the most representative proteins and proteomic profiles of saliva and plasma from patients with fibroadenoma (FA) and infiltrating ductal carcinoma (IDC) of the breast [239] (Table 3).

**Table 3:** Proteomics-based investigation of dysregulated proteins in diverse liquid biopsies/body fluids.

Body Fluids	Proteomics-Based Techniques	Applications in BC
Blood/plasma/serum	SELDI-TOF MS, MALDI-TOF/TOF MS	Identification of panels of serum biomarkers for BC [49]
	2DE, MALDI-TOF MS	Serum proteomic differences between patients with MBC and healthy controls [204]
	LC-MS/MS	BC grading and subtyping, identification of biomarkers for cell growth, ECM and cell-to-cell communication, energy metabolism and gene transcription, cell death and cancer development, transcription regulation, tumorigenesis and invasion, redox balance, and EMT [205]; secretome of BC CAFs [207]; exosomal BC proteome [211]; exosomal phosphoproteome [213]
Proximal fluid proteomics/nipple aspirate fluid (NAF)/dried NAF spots on Guthrie cards	MALDI-TOF MS [214], LC-MS/MS [66], SELDI-TOF MS [215]	Early BC detection; biomarkers discovery in BC [216]
	nLC-ESI-Q-TOF MS	Early BC screening and subtype classification [219]
Milk	LC-MS/MS	Differential protein pattern between breastfeeding mothers with BC compared with healthy women; identification of putative biomarkers for BC [42]; detection of αS1-casein [221]; EVs proteome identification [224]
Urine	LC-MS/MS	Detection of overexpressed proteins in DCIS samples, early invasive and metastatic BC [32]; progressive changes during BC development in rat model [34]
	MALDI-TOF/TOF, LC-MS/MS	Detection of urinary proteome alterations in HER2 enriched BC [33]
Tears	SELDI-TOF MS [40], MALDI-TOF/TOF [38], LC-MS/MS [39]	Identification of differential biomarker profiles for BC patients compared to healthy controls; identification of dysregulated proteins involved in ECM remodeling [39], host immune system pathways, metabolic regulation [38]
Saliva	LC-MS/MS	Identification of biomarkers for DCIS or HER2/*neu* positive or negative BC [36]
	nLC-Q-TOF MS	Differential immune landscape, molecular transport and signaling pathways between FA and IDC [239]
	MALDI-TOF MS, MALDI-TOF/TOF MS [240]	Identification of new BC biomarkers [240]
	SELDI-TOF MS [231], ESI-TOF MS, MALDI-TOF MS [233], ESI-Orbitrap MS, ESI-Q-TOF MS [234]	Panels of biomarkers for accurate discrimination between BC stages [235] or between BC patients and healthy controls [236]

## 8. Conclusions

As a heterogeneous collection of neoplastic diseases, BC requires accurate identification and characterization panels of candidate protein biomarkers for diagnosis and personalized treatment. MS-based proteomics approaches, either based on LC-MS/MS, MALDI-TOF MS, SELDI-TOF MS, MALDI-TOF/TOF MS, or MALDI MSI, complement traditional pathology specific techniques to examine expression level, modification, and interaction of hundreds to thousands of proteins simultaneously. Proteomics-based identification of dysregulated proteins in BC is essential for the following: discovery of new biomarkers; identification of panels of biomarkers for early BC detection and accurate differentiation between BC subtypes; characterization of new subtypes of BC; characterization and quantitation of post-translational modifications (PTMs) and aberrant protein-protein interactions (PPIs); accurate diagnosis and prognostic information and deciphering of molecular functions, biological processes, and mechanisms through which the dysregulated proteins causes breast tumor initiation, invasion, and treatment resistance. 

## Figures and Tables

**Table 2:** Proteomics-based investigation of the most studied PTMs in breast cancer.

PTMs	References	Proteins	Function and Roles in BC
Phosphorylation	[175]	histone H1 isoforms [132]	Putative biomarker of proliferation BC cells [132]
		YWHAH	BC cell migration [181]
		PKA/BAD	Stemness and survival of BCSCs [182]
		ACAP4	Phosphorylated ezrin and phosphorylated ACAP4 interacts to induce membrane fusion of intracellular tubule-vesicles with the apical membrane; cancer progression and metastasis [199], cell migration, polarity, vesicle trafficking and tumorigenesis, regulation of cell adhesion [200]
		ERα	Critical in development and progression of BC [70]
		MZF1 [180]	Development of aggressive BC, control of genes involved in EMT, lysosome-mediated invasion/metastasis [201]
		TUBG1	Phosphorylation deficiency impairs centrosome construction and microtubules nucleation [186]
		CTTN	Phosphorylated CTTN may play a critical role in promoting breast cancer cell mobility and invasion via actin polymerization [185]
		IκBα	Phosphorylation of NF-κB inhibitor alpha is involved in NF-κB TF activity, regulating apoptosis and necroptosis in BC cells [179]
		FAK autophosphorylation	Activation of FAK-SRC signaling complex that trigger pathways involved in cancer cell migration, invasion, proliferation, death and malignant tumor progression [184]
Glycosylation	[177]	membrane proteins [188], i.e., PD-L1	Potential therapeutic strategies to increase cancer immune therapy efficacy [189]
Acetylation	[173]	nuclear proteins [187], ACAP4 [183]	BC cell migration and invasion [183]
Ubiquitination	[178]	PD-L1	Potential therapeutic strategies to increase cancer immune therapy efficacy [189]
SUMOylation	[172]	MZF1	Transcriptional activation or inactivation [201]

## Data Availability

Not applicable.
